# A Coronary Artery Disease Monitoring Model Built from Clinical Data and Alpha-1-Antichymotrypsin

**DOI:** 10.3390/diagnostics12061415

**Published:** 2022-06-08

**Authors:** Chen-Chi Chang, I-Jung Tsai, Wen-Chi Shen, Hung-Yi Chen, Po-Wen Hsu, Ching-Yu Lin

**Affiliations:** 1 Department of Laboratory Medicine, Taipei City Hospital Heping-Fuyou Branch, Taipei 10027, Taiwan; a0679@tpech.gov.tw; 2 Ph.D. Program in Medical Biotechnology, College of Medical Science and Technology, Taipei Medical University, Taipei 11031, Taiwan; d609108005@tmu.edu.tw; 3 Institute of Biotechnology, National Tsing Hua University, Hsinchu 30013, Taiwan; tt22407@gmail.com; 4 Department of Cardiology, Taipei City Hospital Heping-Fuyou Branch, Taipei 10027, Taiwan; dae28@tpech.gov.tw; 5 Preventive Medical Center, Lo-Hsu Medical Foundation Luodong Poh-Ai Hospital, Yilan 26546, Taiwan; 6 School of Medical Laboratory Science and Biotechnology, College of Medical Science and Technology, Taipei Medical University, Taipei 11031, Taiwan

**Keywords:** coronary artery disease, machine learning, plasma, biomarker

## Abstract

Coronary artery disease (CAD) is one of the most common subtypes of cardiovascular disease. The progression of CAD initiates from the plaque of atherosclerosis and coronary artery stenosis, and eventually turns into acute myocardial infarction (AMI) or stable CAD. Alpha-1-antichymotrypsin (AACT) has been highly associated with cardiac events. In this study, we proposed incorporating clinical data on AACT levels to establish a model for estimating the severity of CAD. Thirty-six healthy controls (HCs) and 162 CAD patients with stenosis rates of <30%, 30–70%, and >70% were included in this study. Plasma concentration of AACT was determined by enzyme-linked immunosorbent assay (ELISA). The receiver operating characteristic (ROC) curve analysis and associations were conducted. Further, five machine learning models, including decision tree, random forest, support vector machine, XGBoost, and lightGBM were implemented. The lightGBM model obtained a sensitivity of 81.4%, a specificity of 67.3%, and an area under the curve (AUC) of 0.822 for identifying CAD patients with a stenosis rate of <30% versus >30%. In this study, we provided a demonstration of a monitoring model with clinical data and AACT.

## 1. Introduction

Cardiovascular disease (CVD) is a universal term for the pathologies of disorders in heart and blood vessels, which can be categorized into four types of disease; these are coronary heart disease (CHD), stroke, peripheral arterial disease, and aortic disease [1]. The global leading cause of death at nearly 32% in 2019 [2], CVD has become a worldwide healthcare issue. Among patients suffering from CVD, heart attack and stroke are the most common causes of death. Owing to the fact that 75% of premature CVD is preventable [3], improving risk factors, such as obesity and diabetes, can help to retard the progression of CVD [4,5]. Within these typical categories, coronary artery disease (CAD), which is caused by atherosclerosis, is one of the most common terms in CVD [6,7]. CAD has become a leading factor of death in developed and developing countries, manifesting symptoms as stable/unstable angina, myocardial infarction (MI) or sudden cardiac death [8,9]. As the progression of CAD may take years or decades, monitoring the development of mild CAD is important.

Starting with the buildup of plaque containing cholesterol, fatty substances, and fibrin in the arteries, the inflammatory system may be triggered and cause the process of atherosclerosis incessantly [7]. Accompanying the acceleration of plaque, the symptoms of atherosclerosis may eventually cause acute coronary syndrome such as acute myocardial infarction (AMI). AMI is considered to be the final stage of CAD, and is caused by the sudden interruption of blood flow to the heart and decreased the oxygen supply to the myocardium [10]. Besides acute coronary syndrome, patients with CAD who have had no recent acute events are often considered as having stable coronary artery disease [11]. Even though therapeutic strategies have been well-developed, data that can reflect the progression of CAD are required to assess the efficiency of treatment [12]. Different types of biomarkers may benefit CVD patients in various ways, such as through risk assessment, diagnosis, and monitoring [13]. Most biomarkers were designed for risk assessment and diagnosis in CVD. For instance, patients with AMI can be identified from unstable angina by troponin I/T or acute pulmonary embolism by d-dimer [14,15,16]. Further, high-sensitivity C-reactive protein (hs-CRP) has been the most extensively studied biomarker for the diagnosis of CAD [17,18]. Besides inflammatory biomarkers, B-type natriuretic peptide (BNP) which simply reflects cardiac function can be used for the diagnosis of heart failure (HF) or cardiac dysfunction [19]. However, only a few studies have addressed the development of a monitoring biomarker for CVD [20]. Hence, it is essential to investigate a proper biomarker for monitoring the development of CAD.

Previous studies have reported that cardiac events may be accompanied by the alteration of protein levels. Among these proteins, SerpinA3, also known as AACT, is one of the members which is considered to be an acute phase protein. Serpins (serine protease inhibitors) are an important superfamily of protease inhibitors in humans; for example, SerpinA1 (antitrypsin) and SerpinA3 (Alpha-1-antichymotrypsin), SerpinA6 (corticosteroid-binding globulin) regulate inflammation response [21]. Besides the function of serine protease inhibitors, AACT has been reported to be involved in inflammation [22]. In addition to the function of AACT in the biological process, it is considered to protect tissue via the inhibition of proteolytic enzymes [23]. By forming a serpin-protease complex, the protease may remain covalently linked to serpin and be trapped at the acyl-intermediate stage of the catalytic cycle [21]. The relation between CAD and AACT has been elucidated in previous studies. Aside from the elevation of AACT in CAD and chronic heart failure patients, it has also been proven to be associated with human atherosclerosis, abdominal aortic aneurysm, and the stabilization of the aorta [22,24,25]. Moreover, AACT has been utilized as one of the risk score factors in predicting cardiac events in patients with stable coronary heart disease [24,25]. Nevertheless, the level of AACT in different stenosis rates of CAD has not been reported yet. Further, incorporating clinical data and AACT levels may improve the accuracy of monitoring progression of CAD. 

In this study, we measured the plasma level of AACT concentration in HCs and CAD patients with stenosis rates of <30%, 30–70%, and >70%. Further, association analysis and the ROC curve analysis were conducted to evaluate the potential of AACT to serve as a diagnostic biomarker or monitoring biomarker. After that, five different machine learning models were applied to incorporate clinical data with AACT levels to discriminate CAD patients with differing severity. Finally, the performance of these models was evaluated with the accuracy, precision, f1 score, sensitivity, specificity, and area under the receiving operating characteristic curve (AUC).

## 2. Materials and Methods

### 2.1. Patient Cohort

Our sample set is composed of 198 plasma samples (162 CAD patients and 36 HCs). Plasma samples from 162 patients with CAD (40 female and 122 male patients), and 36 HCs were obtained from the Department of Laboratory Medicine of Taipei City Hospital Heping-Fuyou Branch, and the Cardiovascular Center of Lo-Hsu Medical Foundation Luodong Poh-Ai Hospital. CAD patients were diagnosed by a cardiologist via a coronary angiogram test. Further, patients with acute coronary syndrome (ACS) were not included in this study. Patients with CAD were divided into three classes by coronary artery stenosis rate: <30%, 30~70%, and >70%. This study was approved by the institutional review board of the study hospital, and all volunteers provided informed consent before participating. Patient samples were randomly selected and age-paired with HCs. Clinical and demographic characteristics of CADs and HCs are presented in Table 1. Plasma samples were stored at −80 °C until analyzed. The Taipei City Hospital Research Ethics Committee and the Institutional Review of Cathay General Hospital approved the study protocol (TCHIRB-10910013-E (16 November 2020), CGP-LP106006 (15 June 2017)).

### 2.2. Determination of AACT

To detect AACT levels, 198 plasma samples were examined by enzyme-linked immunosorbent assay (ELISA). Phosphate buffered saline (PBS)-diluted plasma samples (10 μg/mL; 100 μL) or standards (0.1~8 μg/mL) were added to a flat-bottomed 96-well plate and incubated at 37 °C for 2 h. A 3% BSA solution was used for blocking at room temperature for 1 h after washing the plates with PBS containing 0.05% Tween 20 (PBST). The plates were washed with PBST, and a rabbit anti-AACT antibody (Abcam, ab221445, Boston, MA, USA) was added and incubated at 37 °C for 1 h. We washed the plates with PBST, added goat anti-rabbit antibody-horseradish peroxidase (HRP; ab182016, 1:20,000, Abcam, Boston, MA, USA), and incubated the plates at 37 °C for 1 h. After washing the plates, we detected the antibody-HRP with SureBlue Reserve^TM^ TMB Microwell Peroxidase Substrate (Kirkegard & Perry Laboratories, Gaithersburg, MD, USA) and then incubated the plates at room temperature for 30 min. After the color reaction was stopped with 1 N HCl, the absorbance was measured at 450/620 nm. The concentrations of AACT in plasma were measured according to a protein AACT standard curve. Amounts of AACT are expressed as μg/mL. All experiments on samples were analyzed in duplicate.

### 2.3. Statistical Analysis

Description analyses were performed with SPSS 19.0 (IBM, Chicago, IL, USA). The significance of AACT levels between HCs and patients with CAD was determined with a Student’s *t*-test. The Student’s *t*-test was calculated with GraphPad Prism (v.5.0; GraphPad Software, San Diego, CA, USA). The association analysis was calculated using SAS (v.9.3; SAS Institute, Cary, NC, USA). Logistic regression models were used to estimate multivariate-adjusted odds ratios (ORs) and their 95% confidence intervals (CIs) for patients with different stenosis risk rates of CAD. The cut-off values were set as 25th percentile in AACT. The ROC curve analysis and one-way analysis of variance (ANOVA) were conducted with MedCalc (12.4.0.0, Ostend, Belgium). The significance level of all statistical tests was set to *p* < 0.05. The models we built in this study were based on a decision tree (DT), random forest (RF), support vector machine (SVM), extreme gradient boosting (XGBoost), and light gradient boosting machine (lightGBM) with 5-fold cross validation with scikit-learn (vers. 0.21.3). The parameter was tested for optimizing. For DT, the initial value of tree depth was set from 1–10 with a step of 1. The kernel of model was set to entropy or gini. For RF, the initial value of tree number was set from 100 and increased by 100 until 500. The kernel of the model was set to Gini or entropy. For the SVM, the initial value of gamma was set from 10^−6^ to 10^−10^ with a step of 0.1. The initial value of C was set from 10^4^ to 10^7^ with a step of 10. The kernel of the SVM was set to RBF. For XGBoost, the initial value of eta was set from 0.01 to 0.2 with a step of 0.05. The initial value of depth was set from 1 to 10 with a step of 1. As for lightGBM, the initial value of leaves was set from 50 to 400 with a step of 50. The initial value of depth was set from 1 to 10 with a step of 1. The parameters were optimized for the whole dataset and selected by the highest accuracy. A confusion matrix was used in this study to calculate the accuracy, precision, sensitivity, specificity, and f1 score. The value of AUC was calculated from scikit-learn (vers. 0.21.3). The comparison of models was evaluated by ANOVA test.

## 3. Results

### 3.1. Clinical Demographics from Patients with CAD

The characteristics, demographics, and lipid profiles of HCs and CAD patients with stenosis rates of <30%, 30–70%, and >70% were summarized in Table 1. We discovered that the characteristics were significantly different between HCs and patients with CAD, including age, hypertension, hyperglycemia, diabetes, smoking, total cholesterol, HDL-C, and LDL-C. Further, among patients with CAD, sex, ESRD, and TG were found to be significantly different in CAD patients with stenosis rates of >70%. 

### 3.2. Determination of AACT Level in HCs and Patients with CAD

**Figure 1:**
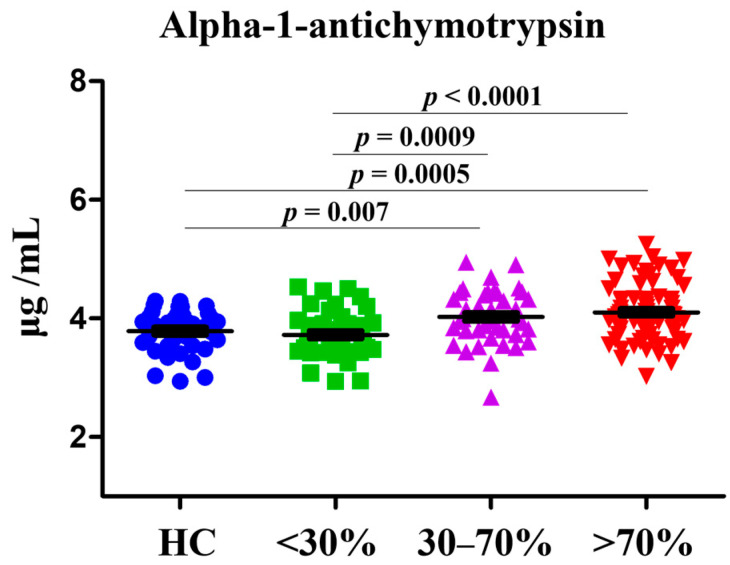
Dot plot of plasma AACT concentrations in healthy controls (HCs), CAD patients with stenosis rates <30%, 30–70%, and >70%.

We examined the plasma level of AACT in HCs and patients with CAD (Figure 1). Plasma levels of AACT in CAD patients with 30–70% of stenosis rates were higher than HCs (1.06-fold, *p* = 0.007) and CAD patients with <30% of stenosis rates (1.08-fold, *p* = 0.0009). Further, plasma levels of AACT in CAD patients with >70% of stenosis rates were higher than HCs (1.08-fold, *p* = 0.0005) and CAD patients with <30% of stenosis rates (1.1-fold, *p* < 0.0001). The ANOVA analysis was performed and received a *p* value <0.001. Moreover, the ROC curve analysis was conducted to evaluate the potential for serving as a monitoring biomarker among different stenosis rates (Table 2). We found that AUC values were 0.726 (with a sensitivity of 73.47 and a specificity of 63.04) with statistical significance (*p* < 0.0001) only in group of <30% versus 30–70%. We speculated that AACT may discriminate subjects with <30% (HC and <30%) and >30% (30–70% and >70%). Thus, we further grouped CAD patients into stenosis rates of <30% versus >30% and received AUC values of 0.702 (with a sensitivity of 65.87 and specificity of 65.85).

### 3.3. Associations of AACT with CAD in Different Stenosis Rate 

In this study, we applied a logistic regression model adjusted for age and sex to calculate the ORs of AACT levels in the development of CAD (Table 3). In the calculation of ORs, we first analyzed HC and CAD groups separately. For instance, HCs versus CAD patients with <30% of stenosis rate, CAD patients with <30% of stenosis rate versus 30–70%, and CAD patients with 30–70% of stenosis rate versus >70%. The baseline characteristics of each group were provided in Table 1 and Figure 1. The ORs of <30% versus 30–70% were significantly associated with AACT levels (ORs = 4.845, *p* = 0.0013). The significance was found between <30% and 30–70%. Therefore, the patients were further grouped by <30% versus >30% and we then calculated the ORs. As result, ORs of <30% versus >30% were significantly associated with AACT level (ORs = 4.235, *p* = 0.0001).

### 3.4. Incorporating AACT to Identify the Severity of Patients with CAD

**Figure 2:**
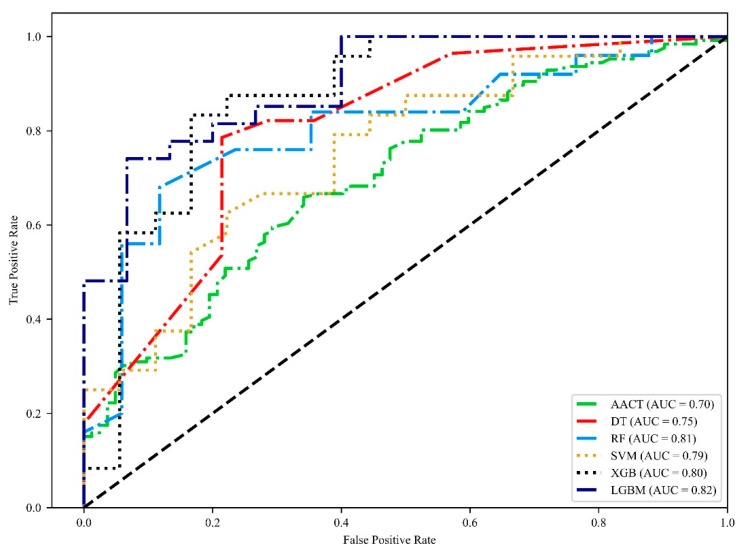
The comparison of ROC plot in AACT and five different models to identify severity of CAD.

**Figure 3:**
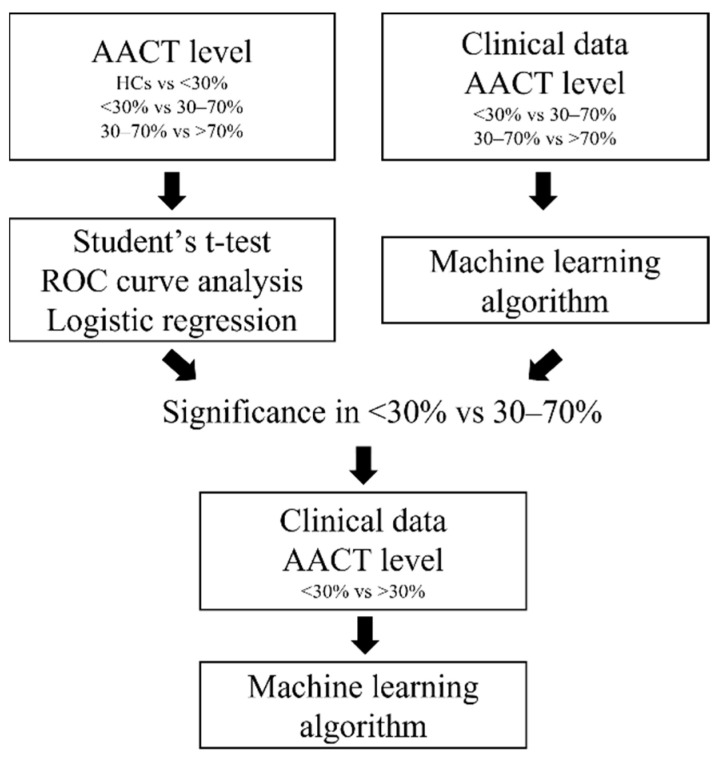
The flowchart of proposed method in this study.

Our goal is to exploit the diagnostic ability of AACT in identifying the progression of CAD. Therefore, after the ROC curve analysis, we incorporated AACT with clinical data we collected such as age, sex, and rates of hypertension, hyperglycemia, diabetes, ESRD, smoking, drinking, total cholesterol, HDL-C, LDL-C, and TG. We first trained and validated the machine learning models without AACT (Table 4). In groups of <30% vs. 30–70%, the models received an accuracy of 42.6% to 48.9%, a precision of 37.5% to 49.1%, an f1 score of 36.6% to 43.7%, a sensitivity of 38.8% to 48.5%, a specificity of 42.3% to 55.7%, and an AUC of 0.442 to 0.52. As groups of <30–70% vs. >70%, the models received an accuracy of 50.8% to 57.3%, a precision of 58% to 66%, an f1 score of 59.1% to 68.8%, a sensitivity of 59.9% to 76.9%, a specificity of 18.1% to 43.2%, and an AUC of 0.466 to 0.571. Next, we trained and validated the machine learning models with AACT (Table 4). In groups of <30% vs. 30–70%, the models received an accuracy of 44.2% to 65.3%, a precision of 39.4% to 63.8%, an f1 score of 36.8% to 60.9%, a sensitivity of 43% to 62.5%, a specificity of 49.8% to 68.7%, and an AUC of 0.486 to 0.669. On the other hand, in groups of 30–70% vs. >70%, the models received an accuracy of 51.2% to 55.8%, a precision of 59.2% to 63.9%, a f1 score 61.1% to 69.3%, a sensitivity of 62.1% to 85%, a specificity of 9.3% to 34.5%, and an AUC of 0.39 to 0.546. We found that AACT significantly improved the accuracy, precision, f1 score, specificity, and AUC in the decision tree, random forest, XGBoost and lightGBM. We further grouped CAD patients into stenosis rates of <30% versus >30%. Finally, in groups of <30% vs. >30%, the models received an accuracy of 70.7% to 76.2%, a precision of 71.8% to 79.1%, an f1 score of 75.8% to 81.5%, a sensitivity of 81.6% to 88.6%, a specificity of 52.5% to 67.3%, and an AUC of 0.751 to 0.822 (Table 5, Figure 2). Among five models, lightGBM received the highest accuracy, precision, and AUC. The proposed method was summarized in Figure 3.

## 4. Discussion

According to our results in this study, we provided a novel strategy to monitor CAD which might help to improve future clinical diagnosis and the onset of treatment. By collecting CAD patient from four cohorts, we were able to compare biological features among patients with different stenosis rates. Next, besides the finding of AACT level upregulation in CAD patients which corresponds to previous results [22,24,25], we also observed a further elevated trend in CAD patients with >30 stenosis rates. Moreover, we conducted machine learning analyses combined with AACT level, stenosis rates, and other biological features mentioned in Table 1. The outcome of these analyses show that the combination of these features may increase the accuracy of monitoring CAD progression. To summarize, this novel finding may bring up a new insight in CAD research.

Although the sample size of our CAD patients from this cohort was sufficient to produce a reliable conclusion, introducing CAD patients from different cohorts may help to strengthen accuracy and reduce bias while analyzing [26,27]. Moreover, different cohorts from different areas may provide some distinctive aspects for our future research. The main risk factors of CAD include obesity, smoking [7], dietary habits, and other lifestyle factors, which can vary depending on location, and may contribute to CAD progression variously [28,29,30]. Therefore, our future studies might focus on the comparison between cohorts from different locations, which may bring cultural effectors into analytical consideration and help to improve the recent analysis strategy.

AACT is a protease inhibitor which involves the regulation of angiogenesis, inflammation, oxidation and fibrotic activities [31]. Li et al. reported that AACT can increase inflammatory factor expression in human umbilical vein endothelial cells (HUVECs) and further regulate vascular smooth muscle cell (VSMC) proliferation and migration [32]. Further, the proliferation and migration of VSMCs are important in atherosclerosis [33]. Moreover, several studies have elucidated the contribution of AACT in cardiac events. For instance, AACT levels have been proven to be upregulated in the patient with AMI and heart failure (HF) group. Zhao et al. investigated the plasma AACT levels in 120 AMI patients and 60 healthy participants. They found that increased circulating levels of AACT in patients with AMI was significantly associated with major adverse cardiovascular events (MACE) [22]. As for HF, Zhao et al. collected epicardial adipose tissue from patients with HF and these were subjected to proteomic analysis. The results showed that among 771 proteins, AACT was highly upregulated in patients with HF [34]. Furthermore, AACT levels have been used to estimate the risk score in CHD patients [24]. In a multicenter study from Ganz et al., AACT level was also considered as a risk in their protein risk score model [24]. In our study, we observed a similar pattern of AACT level elevation in CAD patients (Figure 1). Further, it received AUC 0.77 in identifying CAD patients with <30% and 30–70% (Table 2). Taken together, this evidence suggests that AACT may be used as a biomarker for cardiac events.

We first conducted the analysis with CAD patients grouped by <30% stenosis rate, 30–70% stenosis rate, and >70% stenosis rate. In the association analysis, AACT was found to be significantly associated with CAD patients with 30–70% compared to CAD patients with <30% (Table 3). Moreover, ROC curve analysis showed the best discriminative ability in CAD patients with <30% vs. 30–70% among other groups (Table 2). We then further grouped our samples into <30% (HCs and <30%) versus >30% (30–70% and >70%) and received statistical significance. The results indicated that AACT may serve as a monitoring biomarker between CAD patients with stenosis rates <30% to >30%. To further explore the potential of AACT as a monitoring biomarker, machine learning techniques were applied in our study. Five different models were applied in our experiments. We found that AACT increased the accuracy of all models (Table 4). Therefore, we grouped our samples into <30% versus >30% to train and validate our models. We found that lightGBM showed the highest performance in identifying CAD patients with stenosis rates <30% vs. 30%. The results indicated that AACT contributed to the machine learning discrimination of CAD patients with <30% versus >30%. Moreover, we established a lightGBM model that can identify the severity of CAD with a sensitivity of 81.4%, a specificity of 67.3%, and an AUC of 0.822. In a previous study, Satoh et al. found that an increase of the plasma level of cyclophilin A (CyPA) was associated with the severity of coronary stenosis. They further conducted the ROC curve analysis of CyPA and received an AUC of 0.80 [35]. In another study from Wang et al., they examined a long non-coding (lnc) RNA named AK098656 in patients with coronary heart disease (CHD) and found an increasing level of AK098656 in CHD patients with >70% compared with <70%. However, they only conducted ROC curve analysis in CHD patients against HCs and received an AUC of 0.673 [36]. An elegant study from Ibrahim et al. established a clinical and biomarker scoring system to predict whether patients were suffering from severe coronary stenosis. The model was trained and validated with clinical factors, midkine, adiponectin, apo C-1, and KIM-1. The model received a sensitivity of 77%, a specificity of 84%, and an AUC of 0.87 in discriminating CAD patients with <70% against >70% [37]. Compared to their model, our model has higher sensitivity and provides an earlier diagnosis of coronary stenosis. 

However, several limitations have been addressed in the related work. For instance, a lack of patients with low risk of CAD in the data, machine learning methods which depend on the quality of data, and the requirement for extensive batch-based training [24,38]. Nevertheless, several limitations were encountered in our study. Firstly, more clinical laboratory data should be included to exploit the potential of our models but only risk factors such as lipid profile, drinking, and smoking were collected from the clinic. Secondly, sample size is important to reach a high accuracy and trueness in machine learning. Despite the fact that the results of association analysis, ROC curve analysis, and machine learning models showed the potential of AACT to serve as a monitoring biomarker, the performance of these models should be carefully considered. Lastly, to establish a fully functioning monitoring model for the progression of CAD, more clinical data and biomarkers should be included. Hence, our future work is to enlarge the sample size for each class of stenosis rate and include more biomarkers and clinical data to further optimize our CAD monitoring model. 

## 5. Conclusions

In this study, we discovered that the plasma level of AACT elevation was accompanied by the increasement of coronary stenosis. Furthermore, we observed that AACT is associated with the development of CAD. Lastly, a CAD monitoring model based on lightGBM that can discriminate coronary stenosis at a rate of 30% was established. Our study provided a demonstration of creating a monitoring model with clinical data and AACT.

## Figures and Tables

**Table 1:** Comparison of clinical characteristics and lipid profile between HC and patients with CAD.

	HC	Stenosis Rate of Patients		
		<30%	30–70%	>70%
	n = 36	n = 45	n = 49	n = 68
Age	38.8 ± 10.13	62.89 ± 10.26 **	63.08 ± 9.88 **	62.63 ± 9.20 **
Sex	22 (61.1%)	30 (66.6%)	31 (63.2%)	61 (89.7%) *
Hypertension	1 (2.7%)	24 (53.3%) **	37 (75.5%) **	50 (73.5%) **
Hyperglycemia	0	22 (48.8%) **	30 (61.2%) **	51 (75%) **
Diabetes	1 (2.7%)	8 (17.7%) *	13 (26.5%) **	33 (48.5%) **
ESRD	0	1 (2.2%)	2 (4.08%)	13 (19.1%) **
Smoking	10 (27.7%)	20 (44.4%) *	20 (40.8%) *	40 (58.8%) *
Drinking	9 (25%)	9 (20%)	12 (24.4%)	20 (29.4%)
Use of lipid-lowering agents	-	16 (35.5%)	20 (40.8%)	45 (66.1%)
Total Cholesterol (mg/dL)	186.03 ± 36.67	154.9 ± 31.84 **	154.08 ± 27.68 **	154.13 ± 30.93 **
HDL-C (mg/dL)	54.5 ± 13.95	53.67 ± 19.41	49.22 ± 17.05 *	43.8 ± 15.8 **
LDL-C (mg/dL)	131.08 ± 37.05	95.09 ± 26.94 **	95.43 ± 28.8 **	96.39 ± 31.3 **
TG (mg/dL)	154.5 ± 47.26	146.64 ± 123.52	144.95 ± 103.08	174.12 ± 152.09 *

* means *p* < 0.05, ** means *p* < 0.0001.

**Table 2:** The receiver operating characteristic (ROC) curve analysis of AACT.

	Cut-Off	Sensitivity (95% CI)	Specificity (95% CI)	AUC (95% CI)	*p*-Value
HC vs. 30%	3.743	58.7 (43.2–73)	63.89 (46.2–79.2)	0.598 (0.483–0.704)	0.1303
30% vs. 30–70%	3.810	73.47 (58.9–85.1)	63.04 (47.5–76.8)	0.726 (0.625–0.813)	<0.0001
30–70% vs. 70%	4.027	50.65 (39.0–62.2)	53.06 (38.3–67.5)	0.526 (0.436–0.616)	0.6144
<30% vs. >30%	3.927	65.87 (56.9–74.1)	65.85 (54.6–76)	0.702 (0.635–0.763)	<0.0001

**Table 3:** Association of AACT among HC and CAD patients with stenosis rates of <30%, 30–70%, and >70%.

	Groups		Multivariate Logistic Regression Model	
Cut off	HC (n = 36)	<30% (n = 46)	ORs (95% CI)	*p*-value
3.481	10	22	3.172 (0.625–16.093)	0.1637
	26	24		
	<30% (n = 46)	30–70% (n = 49)	ORs (95% CI)	*p*-value
3.546	22	8	4.845 (1.850–12.691)	0.0013
	24	41		
	30–70% (n = 49)	>70% (n = 77)	ORs (95% CI)	*p*-value
3.800	8	12	1.071 (0.397–2.892)	0.8921
	41	65		
	HC and <30% (n = 82)	>30% (n = 126)	ORs (95% CI)	*p*-value
3.623	32	20	4.235 (2.035–8.816)	0.0001
	50	106		

**Table 4:** Five machine learning models incorporated with and without AACT.

Training and Validating without AACT							
Groups	Models	Accuracy (95% CI)	Precision (95% CI)	f1 Score (95% CI)	Sensitivity (95% CI)	Specificity (95% CI)	AUC (95% CI)
<30% vs. 30–70%	decision tree	48.9% (40.3–57.6%)	49.1% (32.3–66%)	43.7% (32.7–54.8%)	46% (23.8–68.2%)	55.7% (35.7–75.6%)	0.519 (0.412–0.626)
	random forest	44.7% (35.4–54.1%)	37.5% (27.9–47.1%)	36.6% (24.4–48.8%)	38.8% (21.4–56.2%)	51.9% (42.7–61.2%)	0.46 (0.402–0.518)
	SVM	45.3% (37–53.6%)	39.5% (26.6–52.4%)	38.5% (24.6–52.5%)	46.3% (16.2–76.4%)	48.8% (25.6–72%)	0.52 (0.386–0.653)
	XGBoost	42.6% (34.6–50.7%)	47.6% (34.7–60.5%)	41.8% (29.2–54.4%)	41.7% (23.1–60.2%)	48.7% (31.6–65.7%)	0.496 (0.411–0.582)
	lightGBM	44.2% (24.6–63.8%)	41.7% (22.4–61%)	43.2% (23.4–62.9%)	48.5% (23.8–73.2%)	42.3% (16.3–68.3%)	0.442 (0.262–0.622)
30–70% vs. >70%	decision tree	51.5% (42.6–60.5%)	61.4% (51.9–70.9%)	59.1% (47.6–70.6%)	59.9% (41.5–78.3%)	40.4% (26.9–53.9%)	0.531 (0.45–0.611)
	random forest	55.4% (45.9–64.8%)	65.5% (54.6–76.5%)	63.7% (54.7–72.8%)	63.2% (52.2–74.2%)	43.2% (32.2–54.2%)	0.571 (0.507–0.634)
	SVM	56.2% (46.7–65.6%)	62.8% (56.9–68.8%)	68.8% (59.9–77.7%)	76.9% (62.8–91%)	18.1% (9.8–26.3%)	0.466 (0.337–0.596)
	XGBoost	57.3% (50.2–64.4%)	66% (55–76.9%)	68% (62–73.9%)	71.8% (64.5–79.1%)	33% (17.7–48.2%)	0.51 (0.425–0.595)
	lightGBM	50.8% (41.2–60.3%)	58% (48.5–67.4%)	61.9% (52.9–70.9%)	68.6% (53.4–83.7%)	26.1% (11.8–40.3%)	0.483 (0.375–0.592)
**Training and Validating with AACT**							
**Groups**	**Models**	**Accuracy (95% CI)**	**Precision (95% CI)**	**f1 Score (95% CI)**	**Sensitivity (95% CI)**	**Specificity (95% CI)**	**AUC (95% CI)**
<30% vs. 30–70%	decision tree	* 58.4% (47.8–69.1%)	63.8% (46.7–80.9%)	55.5% (42.5–68.5%)	50.9% (36–65.9%)	68.7% (54.8–82.6%)	* 0.631 (0.536–0.725)
	random forest	* 59.5% (50.2–68.8%)	57.5% (44–71.1%)	* 52.9% (43.9–61.9%)	51.8% (38–65.6%)	* 66.8% (50.6–83%)	* 0.604 (0.484–0.724)
	SVM	44.2% (34.2–54.2%)	39.4% (22.2–56.5%)	36.8% (23.7–49.9%)	43% (18.4–67.6%)	49.8% (22.8–76.7%)	0.486 (0.399–0.572)
	XGBoost	58.4% (47.2–69.6%)	* 49.2% (36.6–61.8%)	52% (38.8–65.2%)	58.5% (39.7–77.4%)	* 58.1% (44.1–72.1%)	0.656 (0.547–0.765)
	lightGBM	* 65.3% (53.1–77.5%)	* 63.8% (45.1–82.5%)	60.9% (47.8–74.1%)	62.5% (45.7–79.2%)	68.4% (49–87.8%)	0.669 (0.59–0.748)
30–70% vs. >70%	decision tree	54.2% (46.7–61.8%)	60.4% (50.7–70.1%)	63.6% (55.6–71.6%)	69.3% (56.1–82.5%)	34.5% (21.2–47.7%)	0.516 (0.436–0.597)
	random forest	54.2% (45.5–63%)	61% (52.1–70%)	65.2% (58.5–72%)	73.9% (56.7–91.1%)	29.2% (13–45.4%)	0.546 (0.401–0.691)
	SVM	55.8% (43.6–68%)	59.2% (48.5–69.8%)	69.3% (57.3–81.3%)	85% (68.3–101.7%)	9.3% (1.5–17.1%)	0.39 (0.272–0.507)
	XGBoost	51.2% (42.8–59.5%)	59.6% (48.7–70.5%)	61.1% (53.9–68.2%)	64.4% (54.6–74.2%)	33.6% (15.6–51.5%)	0.505 (0.387–0.622)
	lightGBM	51.9% (42.6–61.2%)	63.9% (52.9–74.9%)	62.4% (51.2–73.6%)	62.1% (48.5–75.7%)	30.2% (17.7–42.6%)	0.466 (0.395–0.537)

* means *p* < 0.05.

**Table 5:** Predictive performance of five models in discriminating CAD patients with stenosis rates <30% and >30%.

Groups	Models	Accuracy (95% CI)	Precision (95% CI)	f1 Score (95% CI)	Sensitivity (95% CI)	Specificity (95% CI)	AUC (95% CI)
<30% vs. >30%	decision tree	70.7% (67–74.4%)	71.8% (62.6–80.9%)	75.8% (71.8–79.8%)	81.9% (74.5–89.3%)	57.2% (45.1–69.3%)	0.751 (0.692–0.809)
	random forest	75.2% (68.4–82.1%)	77.9% (68.7–87.1%)	81.4% (75.2–87.6%)	86.3% (77.2–95.4%)	56% (41.7–70.3%)	0.816 (0.75–0.882)
	SVM	75% (69.5–80.5%)	76% (71.6–80.4%)	81.5% (77.1–85.8%)	88.6% (79.1–98.1%)	52.5% (40.9–64.1%)	0.793 (0.72–0.865)
	XGBoost	75% (69.1–80.9%)	77.1% (71.3–82.8%)	79.3% (73.8–84.9%)	81.8% (75.9–87.6%)	65.1% (58.7–71.6%)	0.805 (0.741–0.87)
	lightGBM	76.2% (71–81.3%)	79.1% (73.2–85.1%)	80.1% (74.4–85.7%)	81.4% (73.1–89.7%)	67.3% (57.7–77%)	0.822 (0.77–0.874)
